# Clinical diagnosis challenges in Zika virus infection

**DOI:** 10.22088/cjim.9.4.416

**Published:** 2018

**Authors:** Mostafa Javanian, Jila Masrour-Roudsari, Soheil Ebrahimpour

**Affiliations:** 1 Infectious Diseases and Tropical Medicine Research Center, Health Research Institute, Babol University of Medical Sciences, Babol, Iran.

**Keywords:** Zika virus, Diagnosis, Flu like illness

**Dear Editor,**

Will Zika virus (ZIKV) infection as a mosquito-borne illness with symptoms similar to flu difficult to diagnose? 

To answer this question, we need to know this infectious disease well and its clinical symptoms. So, first we need to point to the mentioned case. ZIKV is an emerging viral infection in the *Flaviviridae *family, transmitted by *Aedes aegypti*. 

At present according to the reports of health organizations, this infection has spread throughout the USA, the Pacific Islands, and the Southeast Asia. This flu like infection causes mild symptoms resolved in two weeks, like fever, headache, rash, myalgia, redness, and conjunctivitis (1). 

Thus, the temperature is usually low grade fever (within 38.0°C) and skin rashes are likely immune-mediated and pruritic in many cases which begin within 1–4 days onset. Definitely, the complicated features comprising Guillain-Barré syndrome (GBS) and fatal encephalitis in adults, an abnormally small head size (microcephaly) in newborn infants, immune thrombocytopenic purpura (ITP) were documented (2). 

There are many flu like illnesses, which may make clinicians doubtful in the diagnosis of ZIKV infection. Among these diseases can be referred to such terms as *herpes simplex virus* (HSV), acquired immune deficiency syndrome (HIV/AIDS), hepatitis C, Lyme disease, Q fever, dengue fever (DF), measles, and so on.

 In other words, it is confirmed that these common clinical presentations of ZIKV infection performed to be very similar to some arboviral diseases, like as *Chikungunya virus* (CHIKV) and *Dengue virus* (DENV) infection, as a result, a confounding diagnosis. Consequently, a study that was conducted in 2015 revealed 224 dengue cases screened for ZIKV infection, seven patients had positive results for ZIKV infection(3).

 Several in vitro studies suggested cross-reactivity between antibody responses in dengue virus (DENV) as an arthropod‑borne virus and a member of the genus *Flavivirus*, and *Zika virus*(4). Even few research studies suggest that dengue virus enters to cells with Tyro 3, Axl, and Mertk (TAM) and T-cell immunoglobulin and mucin domain (TIM), and these receptors are engaged in ZIKV infection (5). Although other studies showed the differences between these infections that they are very helpful in solving the misdiagnosed problem. 

Rashes in ZIKV infection are more likely to occur in the first week than dengue infection. In the event, rashes regularly appear during recovery phases of dengue disease. Contrary to dengue, hemorrhagic episodes and abnormality in laboratory findings as thrombocytopenia occur less frequently in ZIKV. 

It has shown that different types of edema are more common in ZIKV infections than in DENV illness. In general, people with ZIKV infection, unlike dengue fever, less likely develop severe illnesses and need to be hospitalized. As a consequence, it can be concluded that diagnosis of ZIKV infection and the complete and accurate verification is a great challenge due to low-level viremia and cross- reactivity related to immune system functions. On the other hand, detection of this infection is best during the early-phase, though, diagnosis is seriously problematic at this stage because the disease occurs in this phase asymptomatically (3).

As a result, the best and the most reliable things are the careful evaluation of infection regarding clinical and paraclinical (hematologic) parameters alongside the use of RT-PCR with high specificity and sensitivity as the gold standard for ZIKV detection. Meanwhile, RT-PCR is effective in serum, saliva and semen in 1-2 weeks post infection. Moreover, it is recommended to use acute and recuperating samples for better diagnosis (5).

The use of molecular tests such as Trioplex Real-Time RT-PCR (rRT-PCR) by the Centers for Disease Control and Prevention (CDC) is recommended specially for those who have recently traveled to regions with risk of Zika and even show some symptoms of a disease such as Chikungunya virus (CHIKV) and DENV(5).
